# Mechanisms of gastrointestinal toxicity in neuromyelitis optica spectrum disorder patients treated with mycophenolate mofetil: insights from a mouse model and human study

**DOI:** 10.1128/spectrum.04307-23

**Published:** 2024-06-25

**Authors:** Gong Li, Li-Juan Xia, Ya-Qing Shu, Lei Wan, Qiao Huang, Xiao-Yu Ma, Hai-Yi Zhang, Zi-Jian Zheng, Xi-Ran Wang, Shi-Ying Zhou, Ang Gao, Hao Ren, Xin-Lei Lian, Dan Xu, Sheng-Qiu Tang, Xiao-Ping Liao, Wei Qiu, Jian Sun

**Affiliations:** 1 Lingnan Guangdong Laboratory of Modern Agriculture, National Risk Assessment Laboratory for Antimicrobial Resistance of Animal Original Bacteria, South China Agricultural University, Guangzhou, China; 2 Guangdong Provincial Key Laboratory of Utilization and Conservation of Food and Medicinal Resources in Northern Region, Henry Fok School of Biology and Agriculture, Shaoguan University, Shaoguan, China; 3 Guangdong Provincial Key Laboratory of Veterinary Pharmaceutics Development and Safety Evaluation, South China Agricultural University, Guangzhou, China; 4 Department of Neurology, The Third Affiliated Hospital of Sun Yat-sen University, Guangzhou, China; 5 Department of Neurology, Zhaoqing No. 2 People’s Hospital, Zhaoqing, China; Nanjing Agricultural University, Nanjing, China

**Keywords:** neuromyelitis optica spectrum disorders, mycophenolate mofetil, gastrointestinal toxicity, multi-omics, colitis

## Abstract

Mycophenolate mofetil (MMF) is commonly utilized for the treatment of neuromyelitis optica spectrum disorders (NMOSD). However, a subset of patients experience significant gastrointestinal (GI) adverse effects following MMF administration. The present study aims to elucidate the underlying mechanisms of MMF-induced GI toxicity in NMOSD. Utilizing a vancomycin-treated mouse model, we compiled a comprehensive data set to investigate the microbiome and metabolome in the GI tract to elucidate the mechanisms of MMF GI toxicity. Furthermore, we enrolled 17 female NMOSD patients receiving MMF, who were stratified into non-diarrhea NMOSD and diarrhea NMOSD (DNM) groups, in addition to 12 healthy controls. The gut microbiota of stool samples was analyzed using 16S rRNA gene sequencing. Vancomycin administration prevented weight loss and tissue injury caused by MMF, affecting colon metabolomes and microbiomes. Bacterial β-glucuronidase from Bacteroidetes and Firmicutes was linked to intestinal tissue damage. The DNM group showed higher alpha diversity and increased levels of Firmicutes and Proteobacteria. The β-glucuronidase produced by Firmicutes may be important in causing gastrointestinal side effects from MMF in NMOSD treatment, providing useful information for future research on MMF.

**IMPORTANCE:**

Neuromyelitis optica spectrum disorder (NMOSD) patients frequently endure severe consequences like paralysis and blindness. Mycophenolate mofetil (MMF) effectively addresses these issues, but its usage is hindered by gastrointestinal (GI) complications. Through uncovering the intricate interplay among MMF, gut microbiota, and metabolic pathways, this study identifies specific gut bacteria responsible for metabolizing MMF into a potentially harmful form, thus contributing to GI side effects. These findings not only deepen our comprehension of MMF toxicity but also propose potential strategies, such as inhibiting these bacteria, to mitigate these adverse effects. This insight holds broader implications for minimizing complications in NMOSD patients undergoing MMF therapy.

## INTRODUCTION

Neuromyelitis optica spectrum disorder (NMOSD) is a central nervous system autoimmune disease characterized by inflammation affecting the optic nerve and spinal cord either concurrently or sequentially (1, 2). NMOSD can lead to paralysis, blindness, and potentially respiratory failure (3). Mycophenolate mofetil (MMF) is a commonly utilized immunosuppressant that has demonstrated efficacy in treating NMOSD. However, the use of MMF is often hindered by significant gastrointestinal (GI) side effects, thereby limiting its high-dose and long-term administration (4). Enhanced comprehension of the pathogenesis of GI toxicity associated with MMF could potentially pave the way for novel therapeutic approaches and more efficacious interventions.

MMF is an orally administered prodrug of mycophenolic acid (MPA) known for its efficacy as a reversible, non-competitive inhibitor of inosine monophosphate dehydrogenase within the purine synthesis pathway (5). Its therapeutic benefits primarily stem from its selective inhibition of T- and B-lymphocyte proliferation. The GI toxicity associated with MMF is multifaceted and has been extensively studied using various methodologies, including molecular (6), cellular (7, 8), and whole-animal (5, 9) approaches, in an effort to elucidate the comprehensive disease networks implicated in MMF-induced toxicity. Specifically, research has indicated that the gut microbiota may influence MMF metabolism through the activity of gut bacterial β-glucuronidase (BGUS), which can counteract hepatic glucuronidation, a crucial pathway for xenobiotic elimination. While these studies have yielded valuable findings, the precise mechanisms underlying MMF toxicity in patients with NMOSD remain to be elucidated.

The utilization of sequencing technologies in the fields of medicine and biology has produced data across various levels, encompassing transcriptome, microbiome, and metabolome, collectively constituting a “multi-omics” strategy (10). Recent researchers have shown that the integration of omics data sets can result in a more thorough comprehension of intestinal diseases. For example, a comprehensive examination of gut microbiota and whole transcriptome profiling data utilizing the dextran sodium sulfate/azoxymethane mouse model of inflammatory colorectal cancer indicated enhancements in sphingolipid signaling and the anti-inflammatory lipoarabinomannan biosynthetic pathway (11). Mars et al. identified subset-specific mechanisms associated with irritable bowel syndrome through the analysis of metagenomic and metabolomic data (12). Therefore, multi-omic investigations enable the elucidation of disease mechanisms.

The primary objective of this study was to investigate the mechanism of MMF GI toxicity in NMOSD utilizing a multi-omics approach. Initially, an MMF-induced colitis mouse model was established, followed by the collection of a multi-omic data set through microbiome and metabolome analyses comparing mice with and without colitis. This data set was then utilized to elucidate the associations between metabolite abundance and microbial abundance. The gut microbiomes of NMOSD patients receiving MMF treatment were subsequently analyzed to investigate a potential association between gut microbiomes and MMF-induced gastrointestinal toxicity. These studies collectively offer a comprehensive understanding of the dysregulated signaling pathways involved in MMF-induced GI toxicity and highlight a promising therapeutic strategy for its management.

## MATERIALS AND METHODS

### Strains, chemicals, reagents, and deposited data

The strains, chemicals, and reagents utilized in this study are detailed in Table S1. *Escherichia coli* strains were cultivated under aerobic conditions at 37°C in Luria–Bertani (LB) broth or on LB agar. Anaerobic culturing was exclusively conducted on brain–heart infusion agar or broth as described by Li et al. (13).

### Animal experiments

Female C57BL/6 mice, aged 6 weeks, were subjected to animal experiments conducted at the laboratory animal center of South China Agricultural University in Guangzhou, China. The animals were maintained in accordance with the guidelines for the Care and Use of Laboratory Animals. The mouse feed was enriched with MMF at a concentration of 0.563% (wt/wt), and mouse sampling was conducted in accordance with established protocols (5).

### Histology of mouse tissue

Mice were utilized as the providers of livers, spleens, colon, and cecum, which were subsequently fixed in 4% paraformaldehyde for a period of 24 hours. Subsequently, the organs were embedded in paraffin longitudinally. The tissues were sliced into longitudinal sections measuring 4 µm in thickness, which were then subjected to histological examination through staining with hematoxylin and eosin (H&E).

### Metabolome profiling

The extraction of metabolites, liquid chromatography with tandem mass spectrometry (LC-MS/MS) analysis, data processing, and statistical analysis were conducted according to the methods described by Liu et al. (14) and carried out by Biotree, Shanghai, China. In brief, metabolites present in colon content were analyzed using LC-MS/MS with a 0.3 annotation cutoff. A total of 8,332 peaks were identified, with 372 metabolites remaining after de-noising based on the standard deviation in control colon content samples. The final data set included peak numbers, sample names, and normalized peak areas, which were then imported into the SIMCA 15.0.2 software for further analysis. The data underwent scaling and logarithmic transformation, following the previously described methods (15).

### Microbiome 16S rRNA gene sequencing, alignment, and analysis

Microbial DNA was extracted from fecal samples of patients with NMOSD using the E.Z.N.A. Stool DNA Kit (Omega Bio-tek, Norcross, GA, USA) in accordance with the manufacturer’s protocol. The V3-V4 DNA region was amplified by PCR using the 338F_806R primer set. The PCR amplicons were purified according to the manufacturer’s instructions after extraction from a 2% agarose gel (Axygen Biosciences, Union City, CA, USA). Paired-end sequencing was conducted on an Illumina MiSeq platform, and sequencing, alignment, and analysis were performed by Microeco Tech (Shenzhen, China).

### MALDI-TOF MS^BGUS^ assay

The matrix-assisted laser desorption ionization time-of-flight mass spectrometry (MALDI-TOF MS)^BGUS^ assays were conducted with slight modifications to the established protocol (16, 17). Bacterial cultures were standardized to a uniform concentration, resuspended in 0.9% NaCl solution with MPAG at a final concentration of 100 µg/mL, and incubated at 37°C for 4 hours. Subsequently, the samples were centrifuged at 4,000 × *g* for 5 minutes, and the resulting supernatants were applied onto MSP 384 target polished steel plates (Shimadzu, Kyoto, Japan) and air-dried at room temperature. Following this, 1 µL of the matrix [α-cyano-4-hydroxycinnamic acid (HCCA), Sigma-Aldrich, St. Louis, MO, USA] was applied and allowed to dry at room temperature for 10 minutes. The internal calibration standard for the mass spectrometer was the HCCA polymer ([2M + H]^+^, m/z 379). Mass spectra were collected utilizing a Shimadzu performance mass spectrometer and Shimadzu Biotech MALDI-MS software in positive linear ion mode within the range of 200–900 Da. The parameters set are as follows: ion source 1, 20 kV; ion source 2, 2.62 kV; lens, 6 kV; pulsed ion extraction, 114 ns; electronic gain, enhanced; mode, low range; mass range selection, 300–800 Da; laser frequency, 50 Hz; digitizer trigger level, 2,500 mV; laser attenuator, 25%; laser range, 40%. Seven hundred fifty shots were acquired per position for each spectrum. Peak ratios for MPA([M + Na]^+^) at m/z 343 ± 1.0 and MPAG([M + Na]^+^) at m/z 519 ± 1.0 were used to assess BGUS activity (18).

The *ex vivo* procedure utilized in this study involved the utilization of freshly collected fecal pellets from mice and live gut bacteria obtained through mechanical homogenization of extracted intestinal segments, as detailed in a previous study (19). The assays included the incorporation of 4% (vol/vol) live gut bacteria, which were subsequently subjected to anaerobic incubation with 100 µM vancomycin (VAN) using microbiome profile (MiPro) media to simulate an *in vivo* microbial environment (13). The control group received double-distilled water as the vehicle control. The specimens underwent a 20-hour incubation period, followed by centrifugation of the cultured gut microbiome samples at 4,000 × *g* for 1 minute. Subsequently, the samples were washed twice with 0.9% NaCl solution and resuspended in 90 µL of the same solution. Following this, 10 µL of MPAG at a concentration of 100 µg/mL was introduced to the gut microbiome samples and incubated for an additional hour. Following centrifugation at 4,000 × *g* for 5 minutes, the supernatants were subsequently removed, and 1 µL of the samples was utilized for MALDI-TOF MS analysis. All experiments were conducted using three independent bacterial cultures.

### Cell culture and experimental design

The murine colon cancer cell lines (MC38) were cultured in culture flasks at 37°C with 5% CO_2_ and 95% humidity in dulbecco's modified eagle medium (DMEM) supplemented with 10% fetal bovine serum (FBS) and 1% penicillin/streptomycin, following standard methods outlined by Qasim et al. (20). MC38 cells were established and treated with MPA at concentrations of 5, 10, and 50 µM, or DMSO (control), for 72 hours. Subsequently, real-time PCR, Western blot, and immunofluorescence experiments were conducted.

### Expression analysis

Total RNAs were extracted from MC38 cells in each experimental group utilizing the Trizol method as outlined in a previous study (20). Subsequently, 1 µg of RNA from each group was reverse-transcribed into cDNA for gene expression analysis via real-time PCR. The housekeeping gene GADPH was chosen as the internal reference. Data analysis was conducted using the comparative Ct method, with primer sequences provided in Table S2.

### Western blot

Whole-cell lysates were prepared from each group using protein lysis buffer, and the protein concentration was quantified using a bicinchoninic acid kit. Subsequently, 20 µg of protein sample was subjected to electrophoresis on a 10% sodium dodecyl sulfate-polyacrylamide gel. Following the transfer of the samples onto a polyvinylidene difluoride membrane (Millipore), they were blocked with 5% skim milk in 1× Tris-buffered saline with Tween for 1 hour at room temperature. Subsequently, the membranes were exposed to monoclonal rabbit anti-mouse antibodies (GAPDH, 1:2,000; CLDN2, 1:1,000) overnight at 4°C, followed by incubation with horseradish peroxidase (HRP) -conjugated secondary antibody (1:5,000) for 1 hour. The signals were visualized using an ECL chemiluminescent kit.

### Immunofluorescence assay

MC38 cells were evenly distributed in 24-well plates and exposed to either MPA or DMSO for a period of 72 hours. Subsequently, the cells were fixed in a freshly prepared solution of 4% paraformaldehyde in phosphate buffered saline (PBS) for a duration of 15 minutes. Following permeabilization with 0.5% Triton X-100 in PBS for 15 minutes and blocking with 10% goat serum at 37°C for 1 hour, the sections were subjected to overnight incubation with primary antibody (CLDN2, 1:200) at 4°C. After rinsing with PBS, the sections underwent a 30-minute incubation with a 488-conjugated secondary antibody at room temperature. The results were then examined using laser scanning confocal microscopy (Leica, TCS SP8).

### Patient enrollment

A total of 17 female patients diagnosed with NMOSD and receiving MMF therapy (0.5 g MMF orally, twice a day for long-term therapy) at the Third Affiliated Hospital of Sun Yat-sen University in Guangzhou, China, were included in this study. The primary inclusion criteria encompassed the following: (i) a clinical diagnosis of NMOSD based on clinical characteristics as outlined by Kalari et al. (21), (ii) patients aged between 30 and 70 years, and (iii) an Expanded Disability Status Scale (EDSS) score not exceeding 8.0. Patients meeting any of the subsequent exclusion criteria were not considered: (i) recent administration of antibiotics or probiotics within the preceding 3 months; (ii) emergency treatment, such as plasmapheresis or intravenous methylprednisolone, for acute recurrence within the previous month; and (iii) concurrent presence of diabetes mellitus, systemic autoimmune diseases, or other neurological disorders.

Stool samples were obtained from female participants (*n* = 12) in both the NMOSD patient and healthy control groups. Clinical data, including follow-up time, relapses, EDSS score, and the presence of diarrhea symptoms, were collected. Subsequently, the 17 individuals were categorized into two groups: the diarrhea NMOSD (DNM) group, characterized by patients with diarrhea symptoms and elevated EDSS scores, and the non-diarrhea NMOSD (NM) group, consisting of patients without diarrhea symptoms.

### Statistical analysis

Statistical analysis and graphing were conducted using unpaired Student’s *t*-test or Mann–Whitney *U* test. Significant differences among three or more groups were assessed using one-way ANOVA with Bonferroni’s multiple comparisons test (Prism 8.0, GraphPad Software Inc., San Diego, CA, USA). Compounds with variable importance in projection values greater than 1.0 and two-tailed Student’s *t*-test *P*-values less than 0 were considered statistically significant. Statistical significance was defined as ^*^P < 0.05, ^**^P < 0.01, and ^***^P < 0.001.

## RESULTS

### Vancomycin effectively prevented GI toxicity associated with MMF

**Fig 1:**
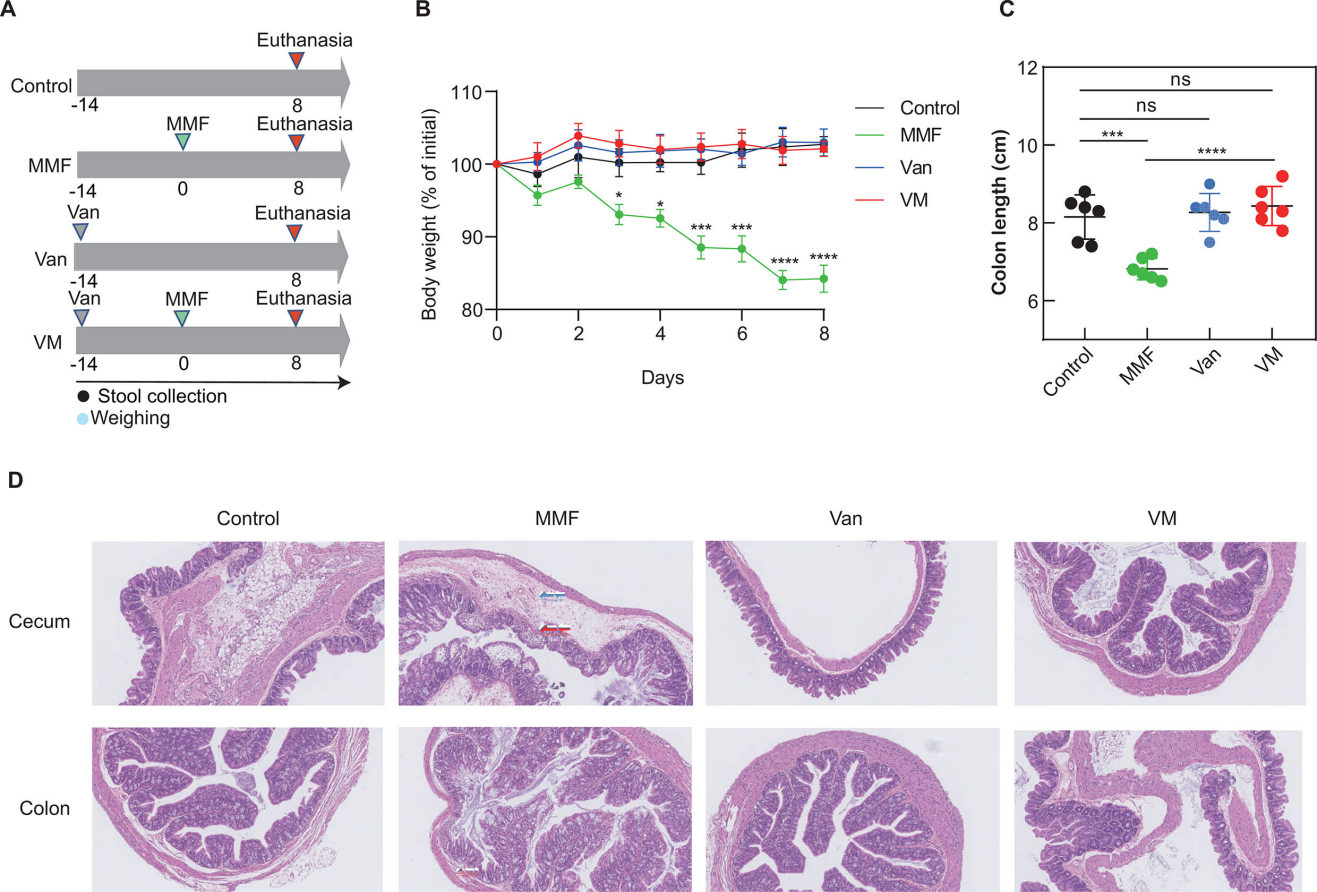
Vancomycin effectively prevented GI toxicity associated with MMF. (**A**) The experimental setup utilized in the MMF mouse model included the administration of MMF, VAN, and a combination of VAN and MMF (VM). (**B**) Mouse body weights. (**C**) Colon length. (**D**) Representative cecum and colon tissues were stained with H&E at 100× magnification for the specified groups. Mild edema in the submucosa was indicated by blue arrows, while inflammatory cell infiltration in tissues was indicated by red arrows. Data are presented as the mean ± standard deviation (*n* = 6 mice per group). Statistical significance was denoted as ^*^P < 0.05, ^**^P < 0.01, ^***^P < 0.001, and ^****^P < 0.0001.

The gut microbiota has been implicated in the development of inflammatory processes in the gastrointestinal tract (11, 22). In this investigation, a bacterial disruption mouse model was employed by administering vancomycin through drinking water (Fig. 1A). It was observed that the body weights of mice in the MMF group significantly decreased compared to the control group, with this effect not observed when vancomycin was pre-administered with MMF (VM group) (*P* < 0.0001) (Fig. 1B). The weights of the heart, liver, spleen, and kidney in mice from the MMF group exhibited a significant decrease when compared to those in the control group (*P* < 0.05). Vancomycin demonstrated a significant preventive effect on these symptoms (*P* < 0.05; Fig. S1). Additionally, vancomycin significantly mitigated the colonic shortening observed in colitis-induced mice treated with MMF (*P* < 0.0001) (Fig. 1C).

The examination of liver and spleen tissue histopathology in control and MMF-treated mice did not show any notable abnormalities (Fig. S2). However, MMF treatment resulted in significant disruption of cecal and colon tissue architecture, characterized by mucosal and submucosal erosion, loss of intestinal crypt structure, and extensive infiltration of inflammatory cells. Co-administration of vancomycin with MMF prevented this tissue damage in the VM group (Fig. 1D). Control mice treated with vancomycin alone did not exhibit significant differences in body weights, cecum and colon tissue destruction, and organ weights compared to untreated control mice. These findings indicate the potential importance of vancomycin in mitigating GI toxicity induced by MMF.

### Vancomycin significantly modified the metabolomic profile of the colon in mice treated with MMF

**Fig 2:**
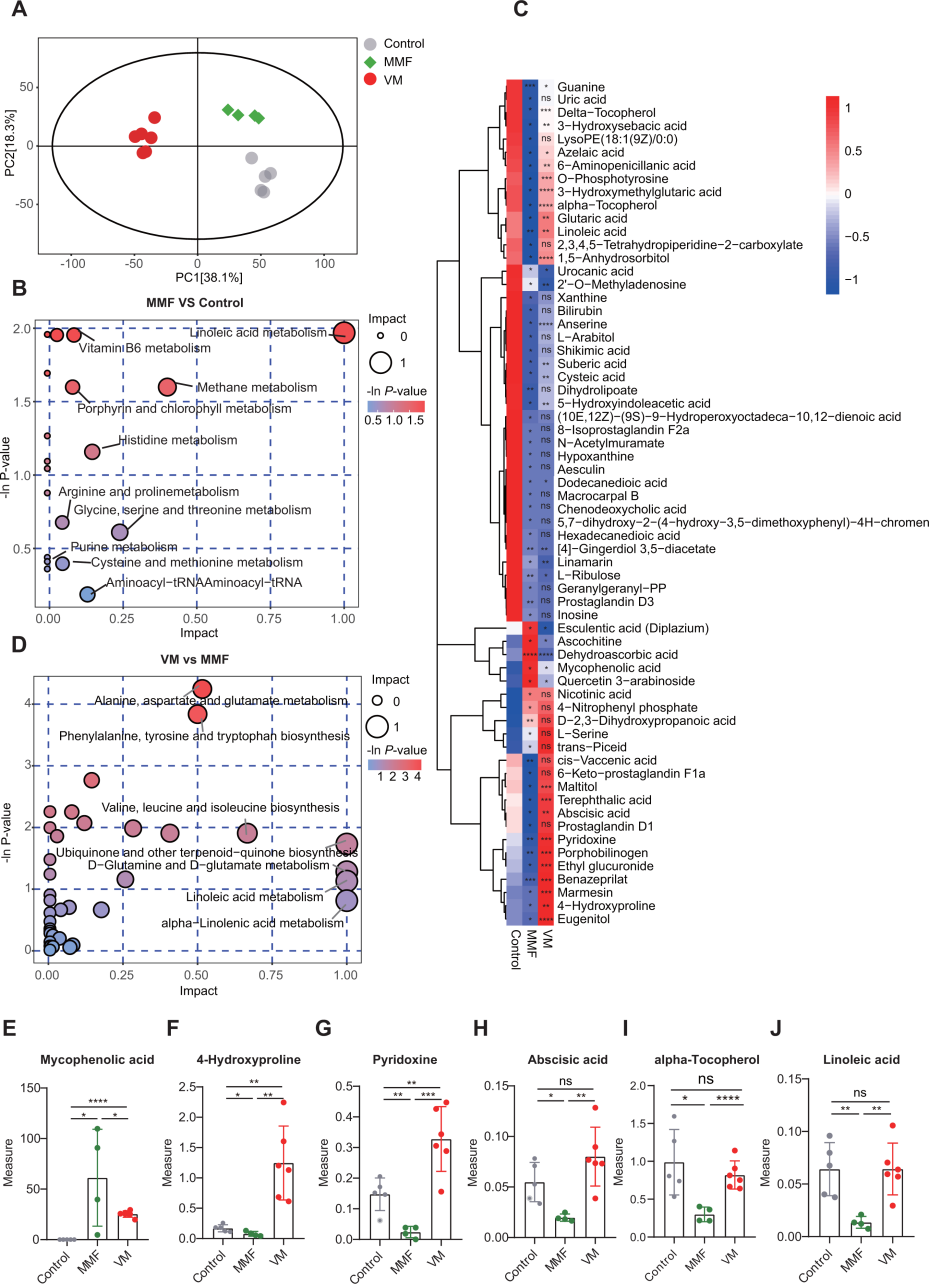
Vancomycin significantly modified the metabolomic profile of the colon in mice treated with MMF. (**A**) PCA of metabolites in the colons of the experimental mice. (**B**) Differential metabolic pathways between the MMF group and control mice. (**C**) Heatmap of the differential metabolites found in colon content samples. (**D**) Differential metabolic pathways between the VM versus MMF mice. The levels of specific metabolites in the colon contents of the indicated mice. (**E**) Mycophenolic acid, (**F**) 4-hydroxyproline, (**G**) pyridoxine, (**H**) abscisic acid, (**I**) α-tocopherol, and (**J**) linoleic acid. *n* = 4–6 mice/group. ^*^P < 0.05, ^**^P < 0.01, ^***^P < 0.001, and ^****^P < 0.0001.

In order to investigate the disparities in metabolic profiles between the MMF and VM groups, we conducted a thorough analysis of colon content samples through metabolomic profiling. Our findings revealed distinct clustering of colon metabolite content among the control, MMF, and VM groups through principal component analysis (PCA). The results suggest that all samples fell within the 95% confidence interval based on Hotelling’s *T*-squared ellipse analysis (Fig. 2A).

Ten metabolic pathways were found to be significantly altered by MMF treatment based on their impact scores. These pathways included linoleic acid metabolism (1.000); methane metabolism (0.400); glycine, serine, and threonine metabolism (0.238); histidine metabolism (0.145); aminoacyl-tRNA biosynthesis (0.129); porphyrin and chlorophyll metabolism (0.083); vitamin B6 metabolism (0.078); arginine and proline metabolism (0.044); purine metabolism (0.043); and cysteine and methionine metabolism (0.025) as shown in Fig. 2B. Furthermore, the analysis identified 64 endogenous metabolites that exhibited significant variations in the colon content samples (Fig. 2C).

Additionally, 23 metabolic pathways demonstrated a high degree of responsiveness to gut bacterial disruption induced by vancomycin. These pathways encompassed ubiquinone and other terpenoid-quinone biosynthesis (1); D-glutamine and D-glutamate metabolism (1); linoleic acid metabolism (1); alpha-linolenic acid metabolism (1); valine, leucine, and isoleucine biosynthesis (0.667); alanine, aspartate, and glutamate metabolism (0.516); and phenylalanine, tyrosine, and tryptophan biosynthesis (0.500) (Fig. 2D). In this categorization, we have delineated 36 metabolites that exhibit differential regulation in response to the perturbation of gut microbiota, such as MPA (Fig. 2E), 4-hydroxyproline (Fig. 2F), pyridoxine (Fig. 2G), abscisic acid (Fig. 2H), α-tocopherol (Fig. 2I), and linoleic acid (Fig. 4J). After exposure to MMF on day 8, elevated levels of MPA were observed in the MMF group (61.21 ± 47.93) compared to controls (0.06 ± 0.03, *P* < 0.05). Pre-administration of vancomycin (Group VM) resulted in a reduction of MPA levels (25.37 ± 3.13, *P* < 0.05). Additionally, levels of 4-hydroxyproline, pyridoxine, abscisic acid, α-tocopherol, and linoleic acid were significantly decreased in the MMF group (*P* < 0.05) and increased in the VM group (*P* < 0.01). These findings suggest that perturbation of gut microbiota leads to changes in metabolite concentrations within the gastrointestinal tract.

### Gut bacteria are required for MMF GI toxicity

**Fig 3:**
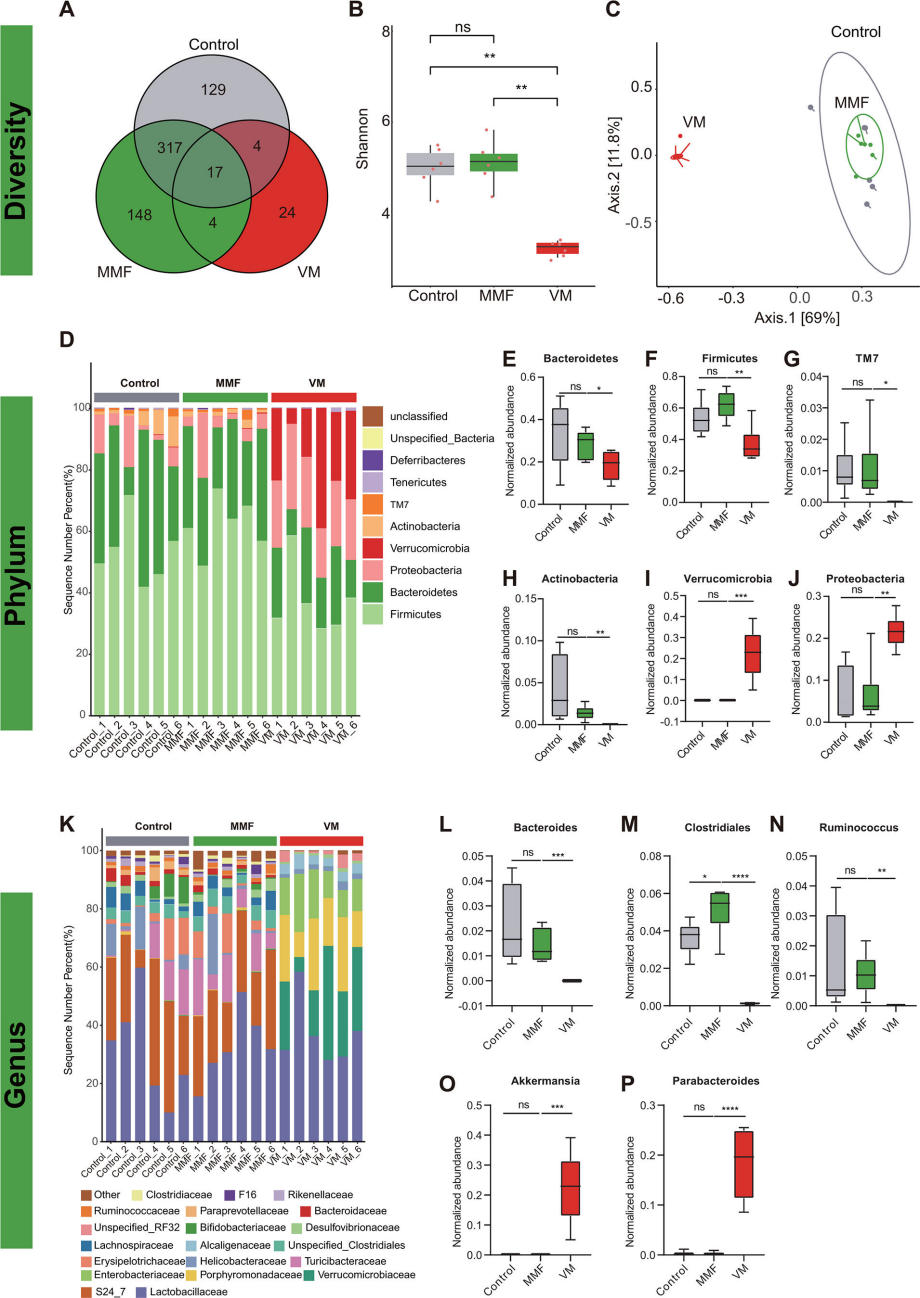
Gut bacteria are required for MMF GI toxicity. Bacterial taxonomic groups in feces from experimental mouse groups. (**A**) OTU and (**B**) Shannon index. (**C**) Unweighted UniFrac PCA of the microbial composition for all groups. (**D**) Phylum level composition of the gut microbiota. Relative abundance of the phyla (**E**) Bacteroidetes, (**F**) Firmicutes, (**G**) TM7, (**H**) Actinobacteria, (**I**) Verrucomicrobia, and (**J**) Proteobacteria. (**K**) Composition at the genus level. (**L**) *Bacteroides*, (**M**) *Clostridiales*, (**N**) *Ruminococcus*, (**O**) *Akkermansia,* and (**P**) *Parabacteroides*. ^*^P < 0.05, ^**^P < 0.01, ^***^P < 0.001, and ^****^P < 0.0001. *n* = 6 mice/group.

The composition of gut microbiota in our experimental mice was determined through the analysis of fecal samples using the 16S rRNA gene sequencing method. The results revealed that 17 operational taxonomic units (OTUs) were present in all control, MMF-treated, and VM groups, while 129, 148, and 24 OTUs were uniquely present in each group, respectively (Fig. 3A). The alpha diversity, as measured by the Shannon index, of the MMF group was comparable to that of the control groups, whereas the VM group displayed lower alpha diversity compared to both the controls and the MMF group (Fig. 3B). Beta diversity was evaluated through PCA on unweighted UniFrac distance matrices, which indicated that the overall composition of the MMF gut microbiota closely resembled that of the control group. Conversely, the VM group led to the development of a distinct phylogenetic microbiota (Fig. 3C).

Analysis at the phylum level did not yield any notable distinctions between the MMF and control groups (Fig. 3D). Conversely, the administration of vancomycin (VM group) resulted in a reduction in the abundance of Bacteroidetes (*P <* 0.05), Firmicutes (*P <* 0.01), TM7 (*P <* 0.05), and Actinobacteria (*P <* 0.01), while facilitating the proliferation of Verrucomicrobia (*P <* 0.001) and Proteobacteria (*P <* 0.01) (Fig. 3F through J).

Significant differences were observed in the abundance of genus *Clostridium* (*P <* 0.05) between the MMF and VM, with the former showing enrichment. Additionally, the abundance of *Bacteroides* (*P <* 0.001), *Clostridium* (*P <* 0.001), and *Ruminococcus* (*P <* 0.01) was notably decreased in the VM group compared to controls. Conversely, *Akkermansia* (*P <* 0.001) and *Parabacteroides* (*P <* 0.0001) showed significant enrichment (Fig. 3K through P). These findings suggest a potential association between gut microbiota composition and MMF-induced GI toxicity.

### Correlation between the microbiota and MPA after MMF treatment

**Fig 4:**
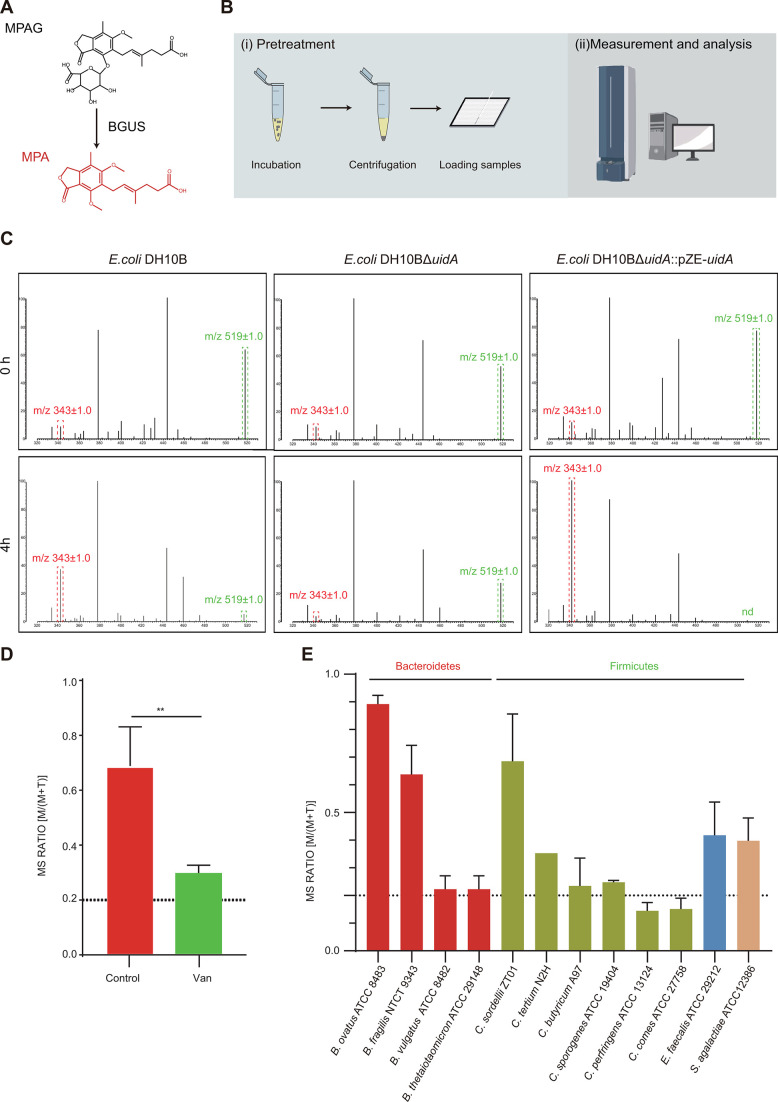
Correlation between the microbiota and MPA after MMF treatment. (**A**) MPAG metabolism by BGUS. (**B**) Experimental flow of the MALDI-TOF MS^BGUS^ assay. (**C**) Representative MALDI-TOF MS spectra of MALDI-TOF MS^BGUS^ assays after 4-hour incubation with DH10B, DH10BΔ*uidA*, and DH10BΔ*uidA*::pZE-*uidA*, respectively. Peaks of MPA (343 ± 1.0 m/z) are indicated by dashed red lines, while peaks of MPAG (519 ± 1.0 m/z) are denoted by dashed green lines. (**D**) Effects of vancomycin on MPAG processing in an *ex vivo* model. (**E**) Comparison of MPAG metabolism abilities among different Bacteroidetes and Firmicutes species. ^**^P < 0.01.

The activities of BGUS have been shown to catalyze the conversion of phenyl-beta-D-glucuronide (MPAG) to MPA, as depicted in Fig. 4A, resulting in GI side effects commonly associated with MMF administration (23). Furthermore, our observations indicate that MPA upregulates the expression of the gut barrier-related *cldn2* gene and CLDN2 protein in a model using MC38 cells, as illustrated in Fig. 4A; Fig. S3A through C. BGUS enzymes possess distinctive active site structures and exhibit unique substrate processing functions, as reported in studies by Little et al. and Ervin et al. (24, 25). Therefore, we posited that only a restricted set of enzymes would have the ability to metabolize MPAG. In order to investigate the primary bacteria involved in MPA metabolism, we conducted an analysis of the associations between the microbiota and MPA at the species level.

Initially, a MALDI-TOF MS^BGUS^ method was developed using MALDI-TOF MS (Fig. 4B) for MPAG metabolism research. Peaks corresponding to MPAG ([M + Na]^+^, 519 ± 1.0 m/z) and the metabolite MPA ([M + Na]^+^, 343 ± 1.0 m/z) were manually identified, and their intensities were documented (Fig. S4). Furthermore, a strain overexpressing the BGUS gene (*uidA*) (*E. coli* DH10BΔ*uidA*::pZE-*uidA*) was introduced to validate the reliability of the method. The findings indicated a reduction in the peak of MPAG and a concomitant elevation in the peak of MPA in both the *E. coli* DH10B and *E. coli* DH10BΔ*uidA*::pZE-*uidA* cohorts. Within the DH10BΔ*uidA*::pZE-*uidA* cohort, the transformation of the substrate (MPAG) into the product (MPA) was complete. Conversely, no analogous occurrence was noted in the *E. coli* DH10BΔ*uidA* group. Subsequently, an *ex vivo* culture model was implemented to confirm the impact of vancomycin on BGUS activity. Our findings indicated that vancomycin decreased the MS ratio [MPA/(MPAG + MPA)] by 2.32-fold compared to the control group (*P <* 0.01, Fig. 4D), providing additional evidence that vancomycin diminishes the MPAG processing capacity of the gut microbiota. Consequently, the MALDI-TOF MS^BGUS^ assay method demonstrates a degree of reliability.

The 16S rRNA gene sequencing analysis revealed the presence of 12 species belonging to the Bacteroidetes and Firmicutes phyla in the microbiomes of mice. Subsequent investigation of these species for their ability to convert MPAG to MPA enzymatically showed that 10 strains exhibited varying degrees of MPAG metabolism (with a range of one- to ninefold increase). However, *C. perfringens* ATCC 13124 and *C. comes* ATCC 27758 were unable to perform this conversion, as illustrated in Fig. 4E. The findings suggest that the presence of Bacteroidetes and Firmicutes members plays a significant role in the toxicity of MMF through the conversion to MPA in the context of metabolic diversity within the bacterial population.

### The gut microbiota of diarrheal NMOSD patients were structurally and compositionally altered

**Fig 5:**
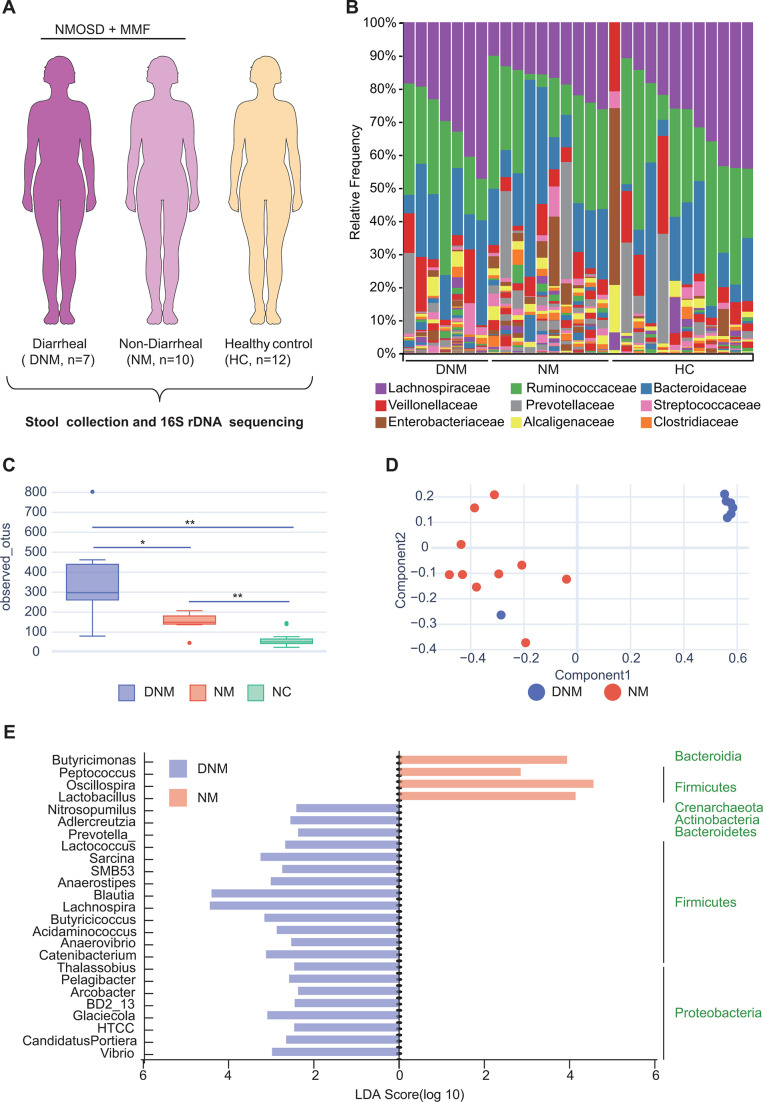
The gut microbiota of diarrheal NMOSD patients were structurally and compositionally altered. (**A**) The study cohort. (**B**) Composition of the gut microbiome from fecal samples at the family level between NMOSD and HCs. (**C**) OTUs observed in the groups. (**D**) Non-metric multidimensional scaling (NMDS) profile of gut microbial diversity. (**E**) LEfSe analysis showing taxa associated with DNM and NM patients. *n* = 7 for DNM group. *n* = 10 for NM group and *n* = 12 for HC group. ^*^P < 0.05. ^**^P < 0.01.

To investigate the potential relationship between MMF-induced diarrhea in NMOSD patients and the composition of their gut microbiota, we conducted a profiling analysis of the gut microbiota in NMOSD patients. Participants were categorized into the DNM or NM groups based on their responses to MMF treatment and were compared to a healthy control group (HC) to assess the potential impact of gut microbiota on diarrhea in NMOSD patients receiving MMF treatment (Fig. 5A).

The microbial composition was assessed through genus-level unsupervised hierarchical OTU clustering, revealing notable distinctions between DNM and NM patients (Fig. 5B). Analysis of microbial alpha diversity, as determined by observed OTUs, indicated significantly higher diversity in the DNM group compared to both the NM (*P <* 0.05) and HC (*P <* 0.01) groups (Fig. 5C). Beta diversity was assessed using NMDS based on Jaccard metrics. The study revealed notable variations in the gut microbial community of MMF-treated NMOSD patients with and without diarrhea, as evidenced by phylogenetic tree-based distances (Fig. 5D). Subsequent analysis at the taxonomic level, using linear discriminant analysis effect size (LEfSe) , identified 21 genera with significant differences in abundance between the two groups. Specifically, *Lachnospira* and *Blautia* were found to be increased in patients with diarrhea, while *Butyricimonas*, *Peptococcus*, *Oscillospira*, and *Lactobacillus* showed decreased levels (Fig. 5D). The primary bacterial phyla observed in DNM samples were Firmicutes and Proteobacteria, as shown in Fig. 5E. These results collectively suggest that there was an increase in alpha diversity, along with a higher abundance of Firmicutes, Proteobacteria, and Bacteroidetes in the DNM group compared to the NM group. Overall, these findings indicate that the gut microbiota’s structure and composition may play a role in influencing the intestinal toxicity of MMF.

## DISCUSSION

MMF has been recognized as a potent immunosuppressant commonly employed in the management of NMOSD and various other medical conditions, yet its therapeutic efficacy is hindered by GI toxicity (26, 27). Existing literature suggests that the GI toxicity of MMF may be attributed to an exaggerated inflammatory reaction and dysbiosis of the gut microbiota, although the precise underlying mechanisms remain elusive (5, 28).

In our previous study, we observed a male-to-female ratio of approximately 1:10 among NMOSD patients in Asian countries, indicating a notably higher prevalence of NMOSD in females compared to males (29). Given the significant impact of gender on gut microbiota composition (30), our study focused on analyzing the gut microbiota of females and utilized female mice as the experimental model. In contrast to our research, Flannigan et al. conducted a study utilizing a male C57BL/6 mouse model and determined that the gut microbiota significantly contributes to MMF toxicity. Hence, this phenomenon should remain consistent regardless of the gender of the mice. The administration of MMF resulted in significant colitis characterized by weight loss, colon shortening, and colonic tissue damage in mice. Notably, treatment with vancomycin mitigated these effects, consistent with previous research findings, thereby implicating the gut microbiome in mediating MMF-induced toxicity (5).

The dextran sulfate sodium-induced colitis model has yielded valuable insights into the metabolic aspects of the gut microbiome disorder (31, 32), whereas the establishment of an MMF-induced colitis model has been less extensive. In our study, we employed non-targeted metabolomics to identify notable variations in gut metabolites resulting from MMF administration, which were found to be modifiable with vancomycin treatment. Specifically, vancomycin was observed to significantly decrease MPA levels while increasing the levels of 4-hydroxyproline, pyridoxine, abscisic acid, α-tocopherol, and linoleic acid in the intestinal environment. A significant amount of research has established a connection between these compounds and the mitigation of colitis (33). For example, patients with ulcerative colitis and Crohn’s disease exhibit notably lower levels of linolenic acid compared to non-inflamed individuals and control subjects (34). These findings align with our MMF-induced colitis model, indicating that the levels of these five distinct metabolites can be influenced by vancomycin treatment, suggesting a correlation with the microbiome.

The microbiome data indicated comparable composition and abundance between the MMF and control groups, with the VM group exhibiting lower Shannon and PCA indices. Specifically, vancomycin administration resulted in decreased abundance of Bacteroides and Clostridiales, which are prominent genera within the gut microenvironment (35). The BGUS activity can catalyze the conversion of the MPA glucuronidated derivative MPAG to release the parent compound MPA within the GI tract (5). MALDI-TOF MS is considered to be a convenient, efficient, expeditious, consistent, and easily obtainable tool in microbiology laboratories when contrasted with alternative mass spectrometry techniques that are combined with gas or liquid chromatography (36, 37). In a prior investigation, a MALDI-Tet(X)-plus method was devised to promptly and dependably identify Tet(X)-producing Gram-negative bacteria (17, 38). Hence, a MALDI-TOF MS^BGUS^ method was developed to investigate the conversion of MPAG to MPA by BGUS enzymes expressed in Bacteroidetes and Firmicutes. This study represents the first direct confirmation of MPAG metabolism by various bacterial species using MALDI-TOF MS. Additionally, a significantly higher microbial richness and diversity were observed in DNM patients compared to NM and HC subjects, suggesting increased BGUS activity in DNM patients. These findings may contribute to elevated MPA levels in the gastrointestinal tract.

The findings of this study offer compelling evidence that bacteria within the Bacteroidetes and Firmicutes phyla, capable of producing BGUS, are able to convert MPAG to MPA, leading to disruptions in intestinal homeostasis and reductions in levels of various metabolites. This process contributes to GI toxicity in patients with NMOSD. Inhibiting BGUS activity using natural and synthetic small molecules has been shown to alleviate GI toxicity induced by irinotecan (CPT11) (39–41). Therefore, the use of BGUS inhibitors may have potential applications in preventing GI toxicity associated with MMF treatment. However, our study was constrained by the need for further experimental validation of BGUS activity in patients with DNM and NM. Additionally, it would be beneficial to include a comparative analysis of the transcriptional levels of the BGUS gene across various species to enhance the credibility of our findings.

### Conclusions

In conclusion, our study demonstrates that the BGUS produced by Bacteroidetes and Firmicutes are capable of metabolizing MPAG into MPA, leading to disturbances in gut homeostasis and contributing to GI toxicity in patients with NMOSD. By conducting a thorough examination of the gut microbiota and intestinal metabolites, we have deduced this interaction and observed similar findings in a mouse model of MMF toxicity. Targeting the inhibition of BGUS may represent a promising strategy for preventing MMF-induced GI toxicity.

## Data Availability

The raw sequence data from the fecal microbiota in this paper were uploaded to the Sequence Read Archive (SRA) data (https://www.ncbi.nlm.nih.gov/sra) under accession number PRJNA976489. The original serum metabolite data were also deposited in the MetaboLights database under accession number MTBLS4392.

## SUPPLEMENTAL MATERIAL

Supplemental material spectrum.04307-23-s0001.docx Fig. S1 to S4; Tables S1 and S2.

ASM does not own the copyrights to Supplemental Material that may be linked to, or accessed through, an article. The authors have granted ASM a non-exclusive, world-wide license to publish the Supplemental Material files. Please contact the corresponding author directly for reuse.
