# Supplementary material for: Mechanisms of gastrointestinal toxicity in neuromyelitis optica spectrum disorder patients treated with mycophenolate mofetil: insights from a mouse model and human study
Source: Microbiol Spectr. 2024 Jun 25;12(8):e04307-23. doi: 10.1128/spectrum.04307-23 (PMC11302255; doi:10.1128/spectrum.04307-23)
Supplement: Supplemental material — Fig. S1 to S4; Tables S1 and S2. [file spectrum.04307-23-s0001.docx]

**Supplementary data**

**Fig S1. Weights of different organs in Control, MMF, Van and VM mice.**

**Fig S2. Representative H&E stained spleen and liver tissues in Control, MMF, Van and VM mice (100× magnification).**

**Fig S3. Influence of tight junction structural protein (CLDN2) on MPA-treated MC38 cells.**

**Fig S4. Representative MALDI-TOF MS spectra of MPA and MPAG.**

**Table S1. Strains, chemicals and reagents used in this study.**

**Table S2. Primers used in this study.**

**
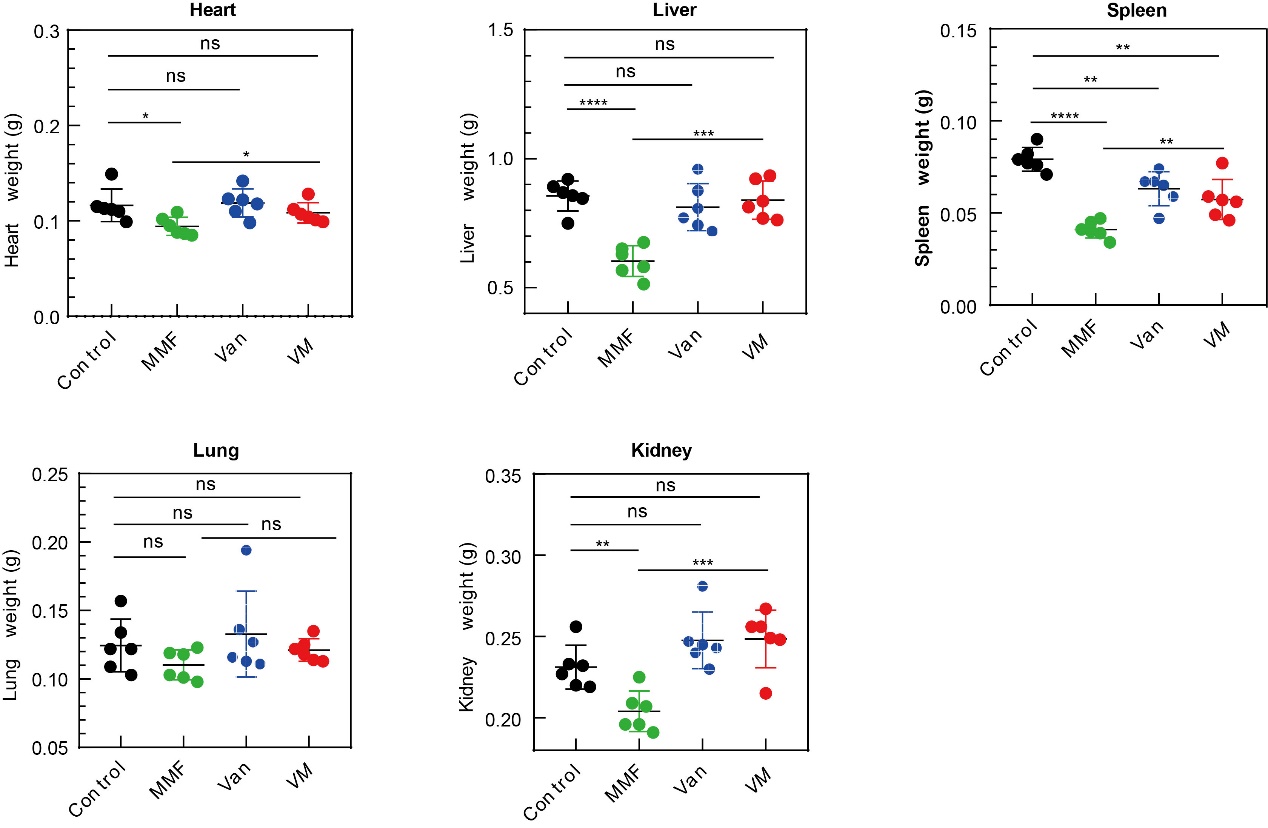
**

**Fig S1. Weights of different organs in Control, MMF, Van and VM mice.**

**
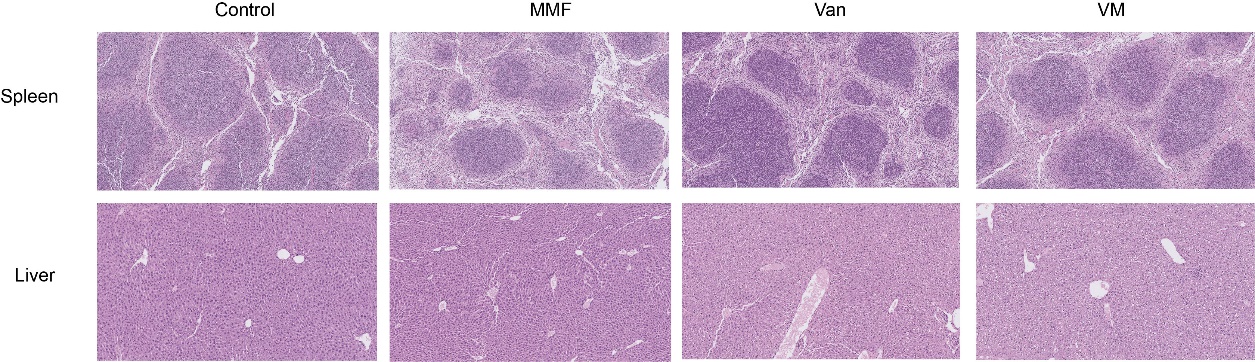
**

**Fig S2. Representative H&E stained spleen and liver tissues in Control, MMF, Van and VM mice (100× magnification).**


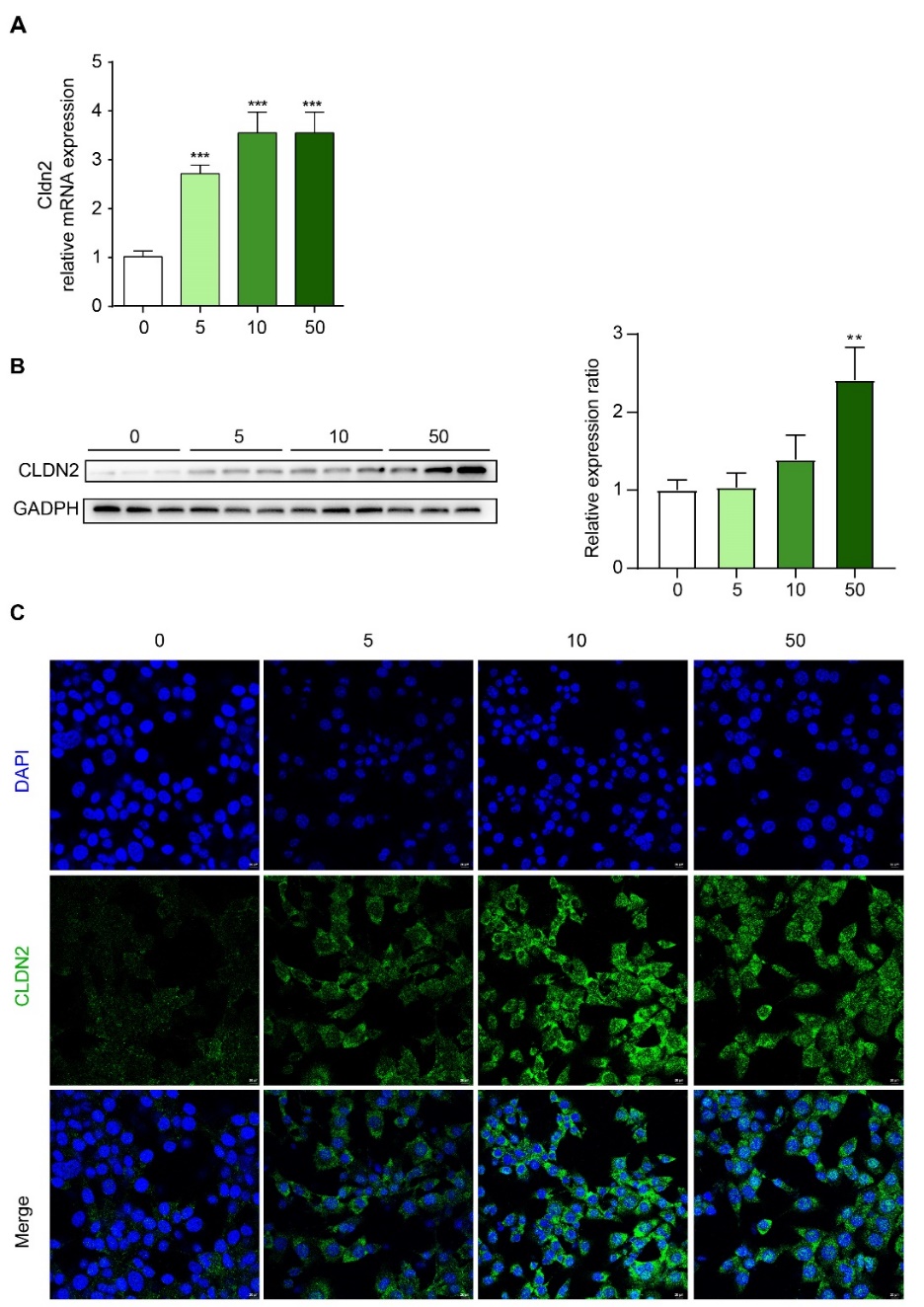


**Fig S3. Influence of tight junction structural protein (CLDN2) on MPA-treated MC38 cells.** (A) The mRNA expression of MC38 cell monolayers treated with either DMSO (Control) or 5 μM, 10 μM, 50 μM MPA. GAPDH served as a housekeeping gene. (B) Western blot and densitometric analysis of CLDN2 in MC38 cell monolayers treated with either DMSO (Control) or 5 μM, 10 μM, 50 μM MPA. (C) Differentiated/polarized monolayers of MC38 cells were treated with either DMSO or 5 μM, 10 μM, 50 μM MPA for 72 hours, fixed, permeabilized, and stained for CLDN2.


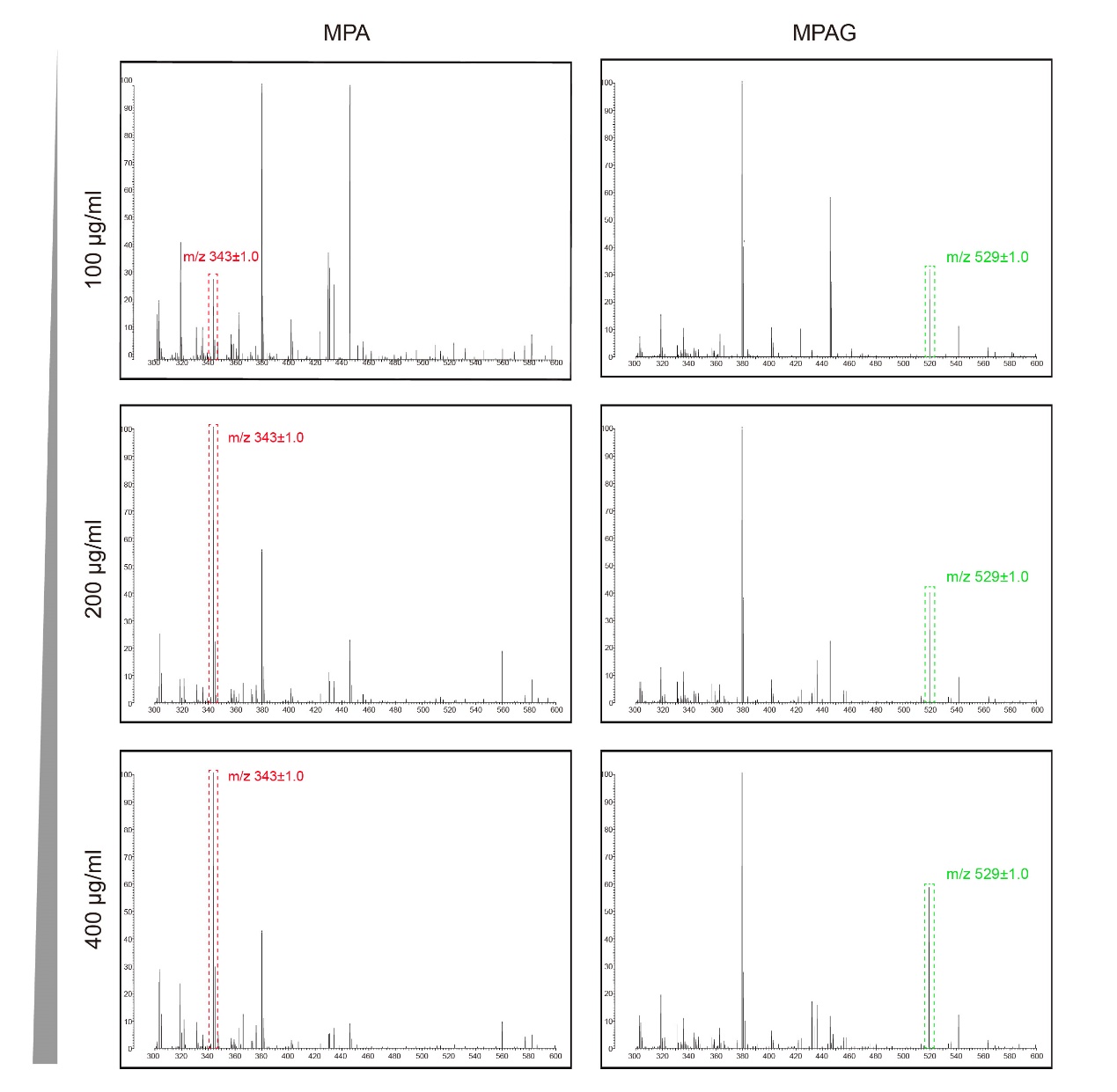


**Fig S4. Representative MALDI-TOF MS spectra of MPA and MPAG.** Peaks of interest are denoted by dashed red lines and represent the MPA peak at 343 ± 1.0 m/z and MPAG at 529 ± 1.0 m/z.

**Table S1. Strains, chemicals and reagents used in this study.**

| **Reagent or Resource** | **Source** | **Identifier** |
| --- | --- | --- |
| **Bacterial strains** | | |
| *E. coli* DH10B^1^ | Tsingke | DH10B |
| *E. coli* DH10BΔ*uidA*^1^ | This study | DH10BΔ*uidA* |
| *E. coli* DH10BΔ*uidA*::pZE-*uidA*^1^ | This study | DH10BΔ*uidA*::pZE-*uidA* |
| *B. ovatus*^2^ | ATCC | ATCC 8483 |
| *B. fragilis*^2^ | NTCT | NTCT 9343 |
| *B. vulgatus*^2^ | ATCC | ATCC 8482 |
| *B. thetaiotaomicron*^2^ | ATCC | ATCC 29148 |
| *C. sporogenes*^3^ | ATCC | ATCC 19404 |
| *C. perfringens*^3^ | ATCC | ATCC 13124 |
| *C. comes*^4^ | ATCC | ATCC 27758 |
| *S. agalactiae*^5^ | ATCC | ATCC 12386 |
| *C. sordellii*^6^ | This study | ZT01 |
| *C. tertium*^3^ | This study | N2H |
| *C. butyricum*^1^ | This study | A97 |
| **Chemicals and reagents.** | | |
| MMF | Macklin | CAS#:115007-34-6 |
| MPA | MCE | CAS#: 24280-93-1 |
| MPAG | TRC | CAS#: 31528-44-6 |
| HCCA | Sigma | CAS#:28166-41-8 |
| BCA kit | Invitrogen | Product code: [A53225](https://www.thermofisher.cn/order/catalog/product/A53225) |
| GAPDH antibody | Proteintech | Product code: 60004-1-Ig |
| CLDN2 antibody | Abcam | Product code: ab53032 |

^1^*Escherichia;* ^2^*Bacteroides*; ^3^*Clostridium;* ^4^*Coporococcus****;*** ^5^*Streptococcus;* ^6^*Clostridium* (*Paeniclostridium*).

**Table S2. Primers used in this study.**

| **Genes** | **Primers** | **Sequence ( 5＇→ 3＇)** | **Reference** |
| --- | --- | --- | --- |
| Cldn2 | cldn2-F | AAGACCACAGCACCAGAG | This study |
|  | cldn2-R | CCACCTCAAGCACAATCC |  |
| *GAPDH* | GAPDH-F | CATCACCATCTTCCAGGAGCG | This study |
|  | GAPDH-R | GAGGGGCCATCCACAGTCTTC |  |
